# Single-photon emission from isolated monolayer islands of InGaN

**DOI:** 10.1038/s41377-020-00393-6

**Published:** 2020-09-09

**Authors:** Xiaoxiao Sun, Ping Wang, Tao Wang, Ling Chen, Zhaoying Chen, Kang Gao, Tomoyuki Aoki, Mo Li, Jian Zhang, Tobias Schulz, Martin Albrecht, Weikun Ge, Yasuhiko Arakawa, Bo Shen, Mark Holmes, Xinqiang Wang

**Affiliations:** 1 State Key Laboratory for Mesoscopic Physics and Frontiers Science Center for Nano-Optoelectronics, School of Physics, Peking University, 100871 Beijing, China; 2 Collaborative Innovation Center of Quantum Matter, 100871 Beijing, China; 3 Electron Microscopy Laboratory, School of Physics, Peking University, 100871 Beijing, China; 4 Institute for Nano Quantum Information Electronics, The University of Tokyo, 4-6-1 Komaba, Meguro-ku Tokyo, 153-8505 Japan; 5 School of Electronic Science and Engineering, University of Electronic Science and Technology of China, Chengdu, 611731 China; 6 Leibniz-Institute for Crystal Growth, Max-Born-Straße 2, 12489 Berlin, Germany; 7 Institute of Industrial Science, The University of Tokyo, 4-6-1 Komaba, Meguro-ku Tokyo, 153-8505 Japan

**Keywords:** Optical materials and structures, Single photons and quantum effects

## Abstract

We identify and characterize a novel type of quantum emitter formed from InGaN monolayer islands grown using molecular beam epitaxy and further isolated via the fabrication of an array of nanopillar structures. Detailed optical analysis of the characteristic emission spectrum from the monolayer islands is performed, and the main transmission is shown to act as a bright, stable, and fast single-photon emitter with a wavelength of ~400 nm.

## Quantum emission: a better way to release single photons

Monolayers of indium gallium nitride (InGaN) sandwiched within isolated nano-pillars composed of gallium nitride (GaN) can be stimulated to emit light in the near-ultraviolet region of the spectrum, one photon at a time. This achievement, by researchers in China, Japan and Germany led by Xinqiang Wang at Peking University, opens a novel route to single-photon emission for research and potential commercial applications. Single-photon emitters are keenly sought for developing new optical quantum computing technologies, including secure communication systems known as quantum key technologies. Several single-photon emission methods already exist, but each is associated with drawbacks, including difficulties with control and stability. The researchers believe their InGaN system could have advantages including wide tunability of the emitted wavelength and compatibility with silicon substrates for manufacturing optoelectronic and power handling devices.

## Introduction

Nonclassical light sources, such as single-photon emitters (SPEs), are essential devices for the realization of future optical quantum technologies, including optical quantum computing and quantum key distribution^1–7^. To date, several strategies have been used to explore the development of SPEs, including the isolation of single atoms^8^, the growth of semiconductor quantum dots (QDs)^9–14^, the use of single molecules^15–17^, and the formation of point defects^18–23^ in wide bandgap and 2D materials^24–28^. Although great strides have been made in the development of solid-state SPEs, including high purity^29^ and indistinguishability^30^ from QDs and high emission rates from both defects and QDs^31^, each technique has its own drawbacks. For instance, point defects form at random locations in their host crystal, tend to have very strong phonon coupling, and exhibit blinking due to metastable states. On the other hand, semiconductor QDs suffer from the fact that difficult-to-control small variations in size can lead to relatively large variations in emission energies from emitter to emitter. Therefore, basic research into the development of SPEs using new materials and techniques is crucial.

In this paper, we present the fabrication and initial optical characterization of a novel type of quantum emitter formed from spatially separated monolayer islands of InGaN sandwiched in a GaN matrix. III-nitride materials are chosen because they are expected to offer several advantages for the development of future devices, including a wide tunability in emission wavelength, compatibility with silicon substrates for growth, and support from a worldwide industrial infrastructure for device fabrication due to their extended use in modern-day optoelectronics and power device applications.

## Results

### Epitaxial growth and structural characterization

The growth procedure of our structures is as follows: first, a 100 nm-thick GaN buffer layer is deposited on a (0001) GaN/sapphire template at 800 °C using molecular beam epitaxy (MBE). Then, in situ evaporation and atomic nitrogen irradiation are adopted to remove any residual metallic Ga on the surface before a monolayer of nominal InN is deposited at a reduced temperature of 650 °C^14^. Finally, a 20 nm-thick GaN barrier is grown on top of the nominal InN layer at the same growth temperature. During the monolayer formation process, island-like regions of InGaN surrounded by GaN form naturally, resulting in the realization of 3-dimensional confinement regions.

**Fig. 1:**
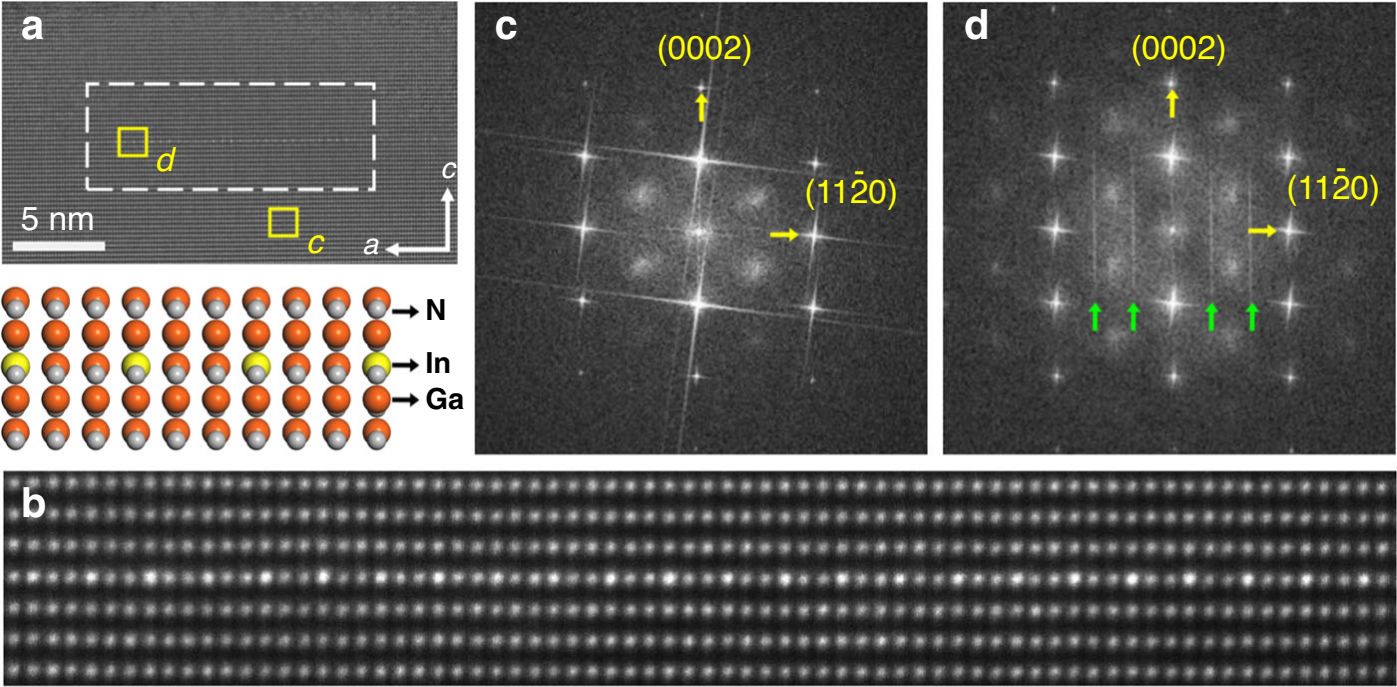
Structural analysis of the monolayer structure. **a** Cross-sectional TEM image of an In(Ga)N/GaN single monolayer island. **b** High-magnification HAADF-STEM image of the In(Ga)N single atomic monolayer, where the top panel shows the corresponding atomic schematic. Fast Fourier transform of the selected areas in (**a**) for **c** the GaN barrier region and **d** the In(Ga)N/GaN monolayer region

Structural analysis of the sample is presented in Fig. 1. In Fig. 1a, we present a typical cross-sectional transmission electron microscopy (TEM) image of an as-grown sample in the $< 1\bar 100 >$ zone axis, showing an InGaN monolayer island (identified by the white dotted rectangle in the image). The islands typically have a lateral size of ~10–20 nm, although some are larger. From the TEM analysis, it is difficult to analyse the areal density of the islands, but we note that they are sometimes laterally separated by up to 50 nm or more. A high-magnification high-angle annular dark-field scanning transmission electron microscopy (HAADF-STEM) image of one of these islands is shown in Fig. 1b, in which the In(Ga)N monolayer is characterized by a periodic intensity variation, with each third atomic column appearing brighter than the surrounding GaN matrix. In the HAADF-STEM image, the brighter and darker spots indicate In- and Ga-rich atomic columns (as shown in the atomic schematic), respectively. We have also investigated the respective reconstructions during growth by RHEED (see Fig. S1), which shows a 1 × 3 reconstruction, interpreted as a $\left( {2\sqrt 3 \times 2\sqrt 3 } \right)R30^\circ$ reconstruction^14^. These results further evidence the periodic arrangement of indium atoms in the TEM image along the $< 11\bar 20 >$ axis, i.e., In:Ga is 1:2.

Fast Fourier transform (FFT) analysis of the TEM image in Fig. 1a at the positions marked as c and d (corresponding to the GaN barrier region and the In(Ga)N/GaN monolayer region, respectively) are shown in Fig. 1c, d. As expected, the FFT pattern of the GaN domain shows a single set of diffraction spots related to the *c*-axis-oriented wurtzite GaN. For the domain with the In(Ga)N monolayer, extra periodical diffraction lines appear along the *c*-axis in reciprocal space, and they divide the original *a*-axis into thirds due to the ordered incorporation of In atoms in the monolayer.

**Fig. 2:**
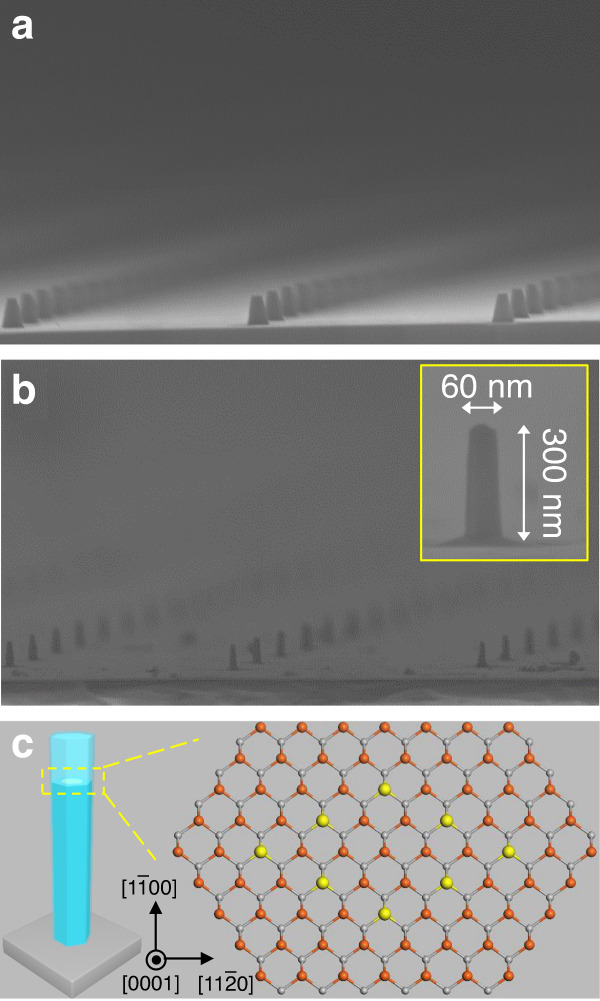
SEM image and schematic representation of a pillar. Tilted-view SEM image of arrays of **a** nanoimprinted In(Ga)N/GaN pillars and **b** after wet etching and regrowth pillars. The inset of the figure presents a typical pillar. **c** Schematic representation of a pillar showing the localized in-plane configuration of In/Ga atoms of the periodic monolayer structure

To facilitate optical mapping of the sample, provide further spatial isolation of individual islands, and increase the photon extraction efficiency, the planar structure was patterned via nanoimprinting lithography and etched into pillars with a 3 μm separation by inductively coupled plasma reactive-ion etching, as shown in Fig. 2a. After an additional wet etching process, the pillars have an average diameter of ~60 nm and a height of ~300 nm (further details of the fabrication process can be found in our previous work^32^). In the final processing stage, an additional GaN conformal shell capping layer of a few nm thickness was grown (also using MBE) to suppress any nonradiative recombination induced by unpassivated surface states along the nanowires^33–35^ and to improve any lateral quantum confinement. The final structure is shown in Fig. 2b, where the inset of the figure presents a typical pillar. Figure 2c shows a more detailed schematic image of a pillar, and the representative atomic model allows us to visualize the localized in-plane configuration of In/Ga atoms of the periodic monolayer structure. Our previous calculations predict that it is the lowest-energy $\left( {2\sqrt 3 \times 2\sqrt 3 } \right)R30^\circ$ In_0.25_Ga_0.75_N configuration^14^.

### Basic optical characterization of the monolayer islands

**Fig. 3:**
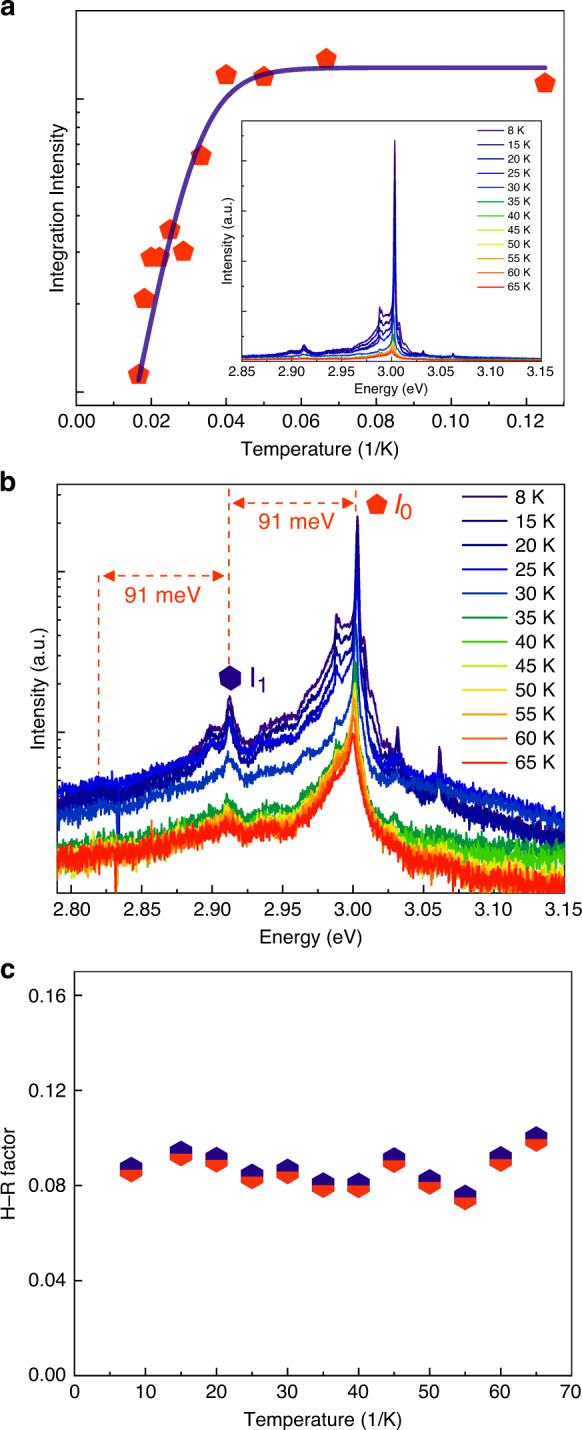
Temperature dependent studies of the monolayer islands. **a** Temperature dependence of the PL integrated intensity from a pillar. The inset shows the spectra measured in the temperature range between 8 and 65K. **b** Temperature-dependent PL spectra on the log scale. **c** Value of the Huang–Rhys factor as a function of temperature

The optical properties of the monolayer islands were investigated using microphotoluminescence (μ-PL) spectroscopy under continuous-wave (CW) excitation at a wavelength of 355 nm (close to the GaN bandgap) and a temperature of 8 K. The emission from individual structures was collected using a ×50 magnification objective lens with a numerical aperture of 0.42 and was analyzed using a 300 mm spectrometer equipped with a liquid-nitrogen CCD camera and a 1200 mm^−1^ reflection grating. Excitation was performed through the objective lens, with a spot size of ~1 μm, such that we could selectively excite individual nanopillar structures. A survey of the pillars (see Fig. 3 for an example emission spectrum) shows the existence of a series of emission lines with distinct narrowband emission with typical linewidths of ~900 μeV, which is comparable to the linewidths of InGaN QD structures in the literature^34,36,37^. Several examples of the emission spectra (from other pillars also measured at 8 K) can be found in the Supplementary Information in Fig. S2a.

The emission spectrum has one main emission peak (hereby labeled *I*_0_), with asymmetric broadening on its low-energy side, likely related to acoustic phonon-related transitions^38^ and possibly emission from other states. The sideband accounts for 30% of the total peak intensity. In addition to the main peak, there is also a low-energy satellite peak, labeled *I*_1_, which can be readily attributed to the LO phonon replica of the main peak, as discussed below. The intensities of all peaks grow linearly with excitation power, indicating their excitonic, rather than biexcitonic, nature (see Fig. S3 in the Supplementary Information).

Figure 3a presents an Arrhenius plot of the normalized integrated PL intensity of peak *I*_0_ of an emitter. The emission is observed to rapidly quench as the temperature is raised beyond 30 K, indicating that the quantum confinement of the structures is weak. The inset of the figure shows the PL spectra as a function of temperature, measured under a constant excitation power of 1 μW.

The PL spectra are presented in log scale in Fig. 3b. As the temperature is raised, the linewidths of the peaks increase due to increased acoustic phonon-related broadening, and the spectrum also exhibits a slight redshift (related to temperature-dependent bandgap shrinkage). The energy separation between *I*_1_ and *I*_0_ is approximately 91 meV, which is in very good agreement with the LO phonon energy in GaN. We also note that, apart from a small absolute shift in energy, all the measured spectra show an almost identical emission structure with the same phonon sideband with more or less the same intensity ratio, strongly indicating that the associated phonon modes are the same, and the emission centers responsible for each spectra have the same structure.

The emission spectrum can be well interpreted using the Huang–Rhys (H–R) model, in which the intensity of the *n*th order phonon replica, *I*_*n*_, is related to that of the zero phonon line, *I*_0_, by $I_n = \frac{{S^n}}{{n!}}I_0$, where *n* represents the number of LO phonons emitted during recombination. The H–R factor, *S*, characterizes the lattice relaxation and is defined as

1 $$S = \mathop {\sum }\limits_q \frac{{\left| {V(q)} \right|^2}}{{E_{LO}}}$$

where *E*_*LO*_ is the LO phonon energy, *V*(*q*) is the matrix element for exciton–phonon interactions, and *q* is the wave vector^39,40^. The matrix element is determined by the ionic property of the lattice and the spatial distribution of the exciton wavefunction, providing a quantitative measurement of the LO phonon coupling strength and, by extension, the degree of the associated emission center’s localization in the host semiconductor^41–43^. From the spectra, we evaluate an H–R factor of 0.1 ± 0.01 (very much in the weak coupling regime) and plot its temperature dependence in Fig. 3c. The H–R factor remains constant as the temperature increases, in agreement with H–R theory^44,45^.

Analysis of *I*_0_ and its sideband is slightly more complicated. Such asymmetric broadening is typically identified as acoustic phonon-assisted recombination in III-nitride nanostructures^38^, but this does not fully explain the emission structure observed in the sideband of the current spectra. As mentioned above, it is possible that the observed sideband consists of a mixture of acoustic phonon-related emission and a series of other emission peaks from transitions at different energies or emission from the same transition undergoing temporal fluctuations due to spectral diffusion^46^ caused by fast (faster than the spectrum acquisition time) fluctuations in the electronic environment around the emitter.

### Operation as a single-photon emitter

**Fig. 4:**
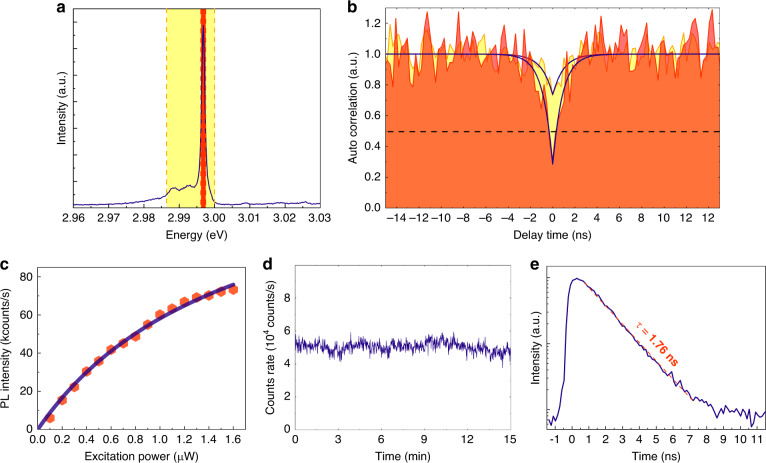
Single-photon nature of the emitter at 8 K under 355 nm excitation. **a** Photoluminescence spectrum from the chosen measurement regions. **b** Autocorrelation of the main peak as the orange shaded rectangle and the lower energy peaks as the yellow shaded rectangle in (**a**). **c** Intensity of the PL emission as a function of the excitation power. The saturation curve yields a saturation power of 1.66 μW and a maximum intensity of 154 kcounts/s. **d** Photon stability measurement at an excitation power of 1 μW over a long time of 15 min. No obvious blinking or spectral diffusion was observed. **e** Lifetime measurement of the emitter fitted with a single exponential yielding a lifetime of ~1.76 ns

To evaluate the nature of the emission, we investigate *I*_0_ and its sideband via measurements of the second-order coherence (intensity autocorrelation) function:

2 $${g}^{\left( 2 \right)}({\uptau}) = \frac{{\langle I(\tau )I(t + \tau )\rangle }}{{I(\tau )^2}}$$

where *τ* is the time delay and *I*(t) is the photoluminescence intensity at time *t*. These measurements were performed using a Hanbury-Brown and Twiss (HBT) setup at 8 K under 355 nm continuous optical excitation with a power of 0.5 μW. The exit slit of the spectrometer was used as a spectral filter with a tuneable bandwidth to filter the emission before measurement. The emission was then directed to the HBT setup, which consisted of a 50/50 beam splitter, two photomultiplier tubes and timing electronics. Figure 4a shows the emission spectrum from our studied emitter. The two shaded regions (orange and yellow) represent different spectral ranges that were selected using the exit slit of the spectrometer. The corresponding autocorrelation traces are presented in Fig. 4b: the autocorrelation trace in yellow corresponds to the broader measurement window with a spectral range of ~13.6 meV, corresponding to a combined measurement of the main peak and the lower energy sideband. The autocorrelation trace in orange corresponds to a spectrally selective measurement of only the brightest emission peak (and some underlying background emission).

While the measurement of the broader window (yellow) exhibits some evidence of antibunching via the clear suppression of counts at *τ* = 0, the value of *g*^(2)^(0) is greater than 0.5, which prohibits the identification of a SPE. However, when the measurement window is narrowed (orange) to include just the main peak (and a small amount of underlying spectral background), the photon statistics exhibit clear and pronounced antibunching with a value of 0.28 ± 0.09, clearly showing the single-photon nature of the emission and hence the isolation of a single quantum transition. Note that this measurement and this analysis have been achieved without any background subtraction or artificial corrections, and we expect to be able to measure single-photon emission with higher purity by improving our experimental setup and using detectors with lower dark count rates. Autocorrelation curves from other corresponding PL spectra in Fig. S2 also show *g*^(2)^(0) < 0.5 (not shown).

Next, to provide some additional characterization of *I*_0_ in terms of performance as a SPE in more detail, we performed several further experiments. First, to analyse the emission brightness, we recorded an emission intensity saturation curve by measuring the PL intensity as a function of the excitation power. The results are shown in Fig. 4c, and the data were fitted with a power model of the following form:

3 $$I_p = I_\infty P/(P + P_{sat})$$

where *I*_*p*_ is the measured intensity count rate, *P* is the excitation power, and *I*_∞_ and *P*_*sat*_ are two fitting parameters: the emission rate and excitation power at saturation, respectively. For this representative emitter, we obtain *P*_*sat*_ = 1.66 ± 0.15 μW and *I*_∞_ = 1.54 ± 0.08 × 10^5^ counts/s. This intensity value is comparable to that of other single emitters in QDs or 2D materials^20,23,47,48^. We note that even with our low excitation power of 0.5 μW, as shown in Fig. 4, we are still able to measure a respectable 3.5 × 10^4^ counts/s, which, after correction for the throughput of our optical system and the detector efficiency, rises to a value of 4.6 × 10^5^ photons per second emitted into the 0.42 NA collection region of the objective lens. Moreover, finite difference time domain (FDTD) simulations of the emission from the structure suggest that we are in fact collecting just ~8% of all emitted photons with our objective lens, and this may be improved by using a higher NA lens or by further design optimization of the nanopillar structure.

Next, the photostability of the source was investigated by measuring successive PL spectra over a period of 900 s using a 1 s acquisition time (data are presented in Fig. 4d). We note that we observe very little spectral diffusion and no obvious blinking or bleaching within the time resolution of the measurement. Such properties are highly beneficial for applications of quantum emitters, where photostability is considered an important factor for practical devices.

Finally, the PL lifetime of the emitter was measured using time-resolved photoluminescence measurements with a frequency-doubled Ti:sapphire pulsed laser emitting at 355 nm (80 MHz repetition rate). The lifetime measurement results at 8 K are shown in Fig. 4e. The data are well described by a monoexponential fit with a lifetime of ~1.76 ns, indicating that the emitter may be used for fast photon emission in the future. The relatively short lifetime in this case is likely due to a relatively high degree of carrier wavefunction overlap in the monolayer structures. Further optical properties and measurement methods are presented in the Supplementary Information.

We note that all emitters that we have measured lie within the relatively narrow energy range of ~150 meV from 2.887 to 3.042 eV. It is likely that the variations in energy arise due to differences in the lateral size of the islands or due to different spatial positions of the islands within the nanowires, where the strain may be different and the overall confinement potential could be modified by surface pinning.

## Discussion

In conclusion, we have demonstrated an approach to create spatially localized and well-separated quantum emission sites in the near-ultraviolet spectral regime using islands of chemically ordered InGaN with monolayer thickness. A detailed analysis of a characteristic emitter proved single-photon generation with *g*^(2)^(0) = 0.28 ± 0.09 and several other properties that are suitable for future applications, such as a fast emission lifetime of 1.76 ns and a high degree of photostability. We have also discussed several possibilities for future improvement of the device.

## Supplementary information

SUPPLEMENTARY INFORMATION FOR Single photon emission from isolated monolayer islands of InGaN
