# Supplementary material for: Single-photon emission from isolated monolayer islands of InGaN
Source: Light Sci Appl. 2020 Sep 9;9:159. doi: 10.1038/s41377-020-00393-6 (PMC7481781; doi:10.1038/s41377-020-00393-6)
Supplement: Supplementary file 1 — SUPPLEMENTARY INFORMATION FOR Single photon emission from isolated monolayer islands of InGaN [file 41377_2020_393_MOESM1_ESM.docx]

**SUPPLEMENTARY INFORMATION FOR**

**Single photon emission from isolated monolayer islands of InGaN**

Xiaoxiao Sun^1^, Ping Wang^1,2^, Tao Wang^3^, Ling Chen^1^, Zhaoying Chen^1^, Kang Gao^4^, Tomoyuki Aoki^4^, Mo Li^5^, Jian Zhang^5^, Tobias Schulz^6^, Martin Albrecht^6^, Weikun Ge^1^, Yasuhiko Arakawa^4^, Bo Shen^1,2^, Mark Holmes^4,7,^*, and Xinqiang Wang^1,2,^*

^1^State Key Laboratory for Mesoscopic Physics and Frontiers Science Center for Nano-optoelectronics, School of Physics, Peking University, Beijing 100871, China

^2^Collaborative Innovation Center of Quantum Matter, Beijing 100871, China

^3^Electron Microscopy Laboratory, School of Physics, Peking University, Beijing 100871, China

^4^Institute for Nano Quantum Information Electronics, the University of Tokyo, 4-6-1 Komaba, Meguro-ku, Tokyo 153-8505, Japan

^5^School of Electronic Science and Engineering, University of Electronic Science and Technology of China, Chengdu, 611731, China

^6^Leibniz-Institute for Crystal Growth, Max-Born-Straße 2, 12489 Berlin, Germany

^7^Institute of Industrial Science, the University of Tokyo, 4-6-1 Komaba, Meguro-ku, Tokyo 153-8505, Japan

*Corresponding author: [wangshi@pku.edu.cn](mailto:wangshi@pku.edu.cn), [holmes@iis.u-tokyo.ac.jp](mailto:holmes@iis.u-tokyo.ac.jp)

1. ***In situ* RHEED pattern after deposition of GaN and InN**

At the end of the GaN barrier growth, the RHEED exhibits a 2×2 surface reconstruction, as shown in Figs. S1a and 1b for the <$1\bar{1}00$> and the <$11\bar{2}0$> azimuth, respectively. Subsequent deposition of InN leads to a 3× periodicity along the <$1\bar{1}00$> azimuth, while a 1× periodicity persists along the <$11\bar{2}0$> azimuth, as shown in Figures S1c and 1d, respectively. These observations are consistent with the HAADF-STEM images, making further evidence the periodical arrangement, i.e., In:Ga to be 1:2 in the monolayer region.


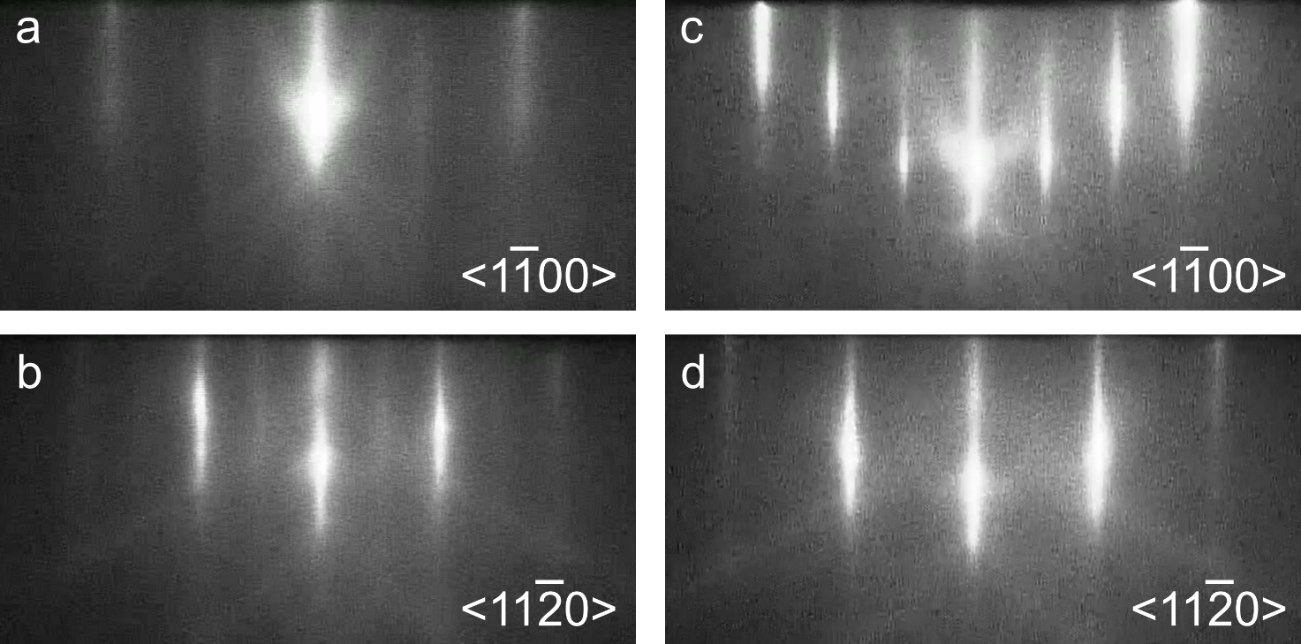


**Figure S1.** In situ RHEED pattern after deposition of a) and b) GaN, as well as c) and d) InN along the <$1\bar{1}00$> and the <$11\bar{2}0$> azimuth, respectively.

1. **Optical properties from four examples of emitters**

Several examples of the emission spectra (from other pillars also measured at 8 K) are shown below.


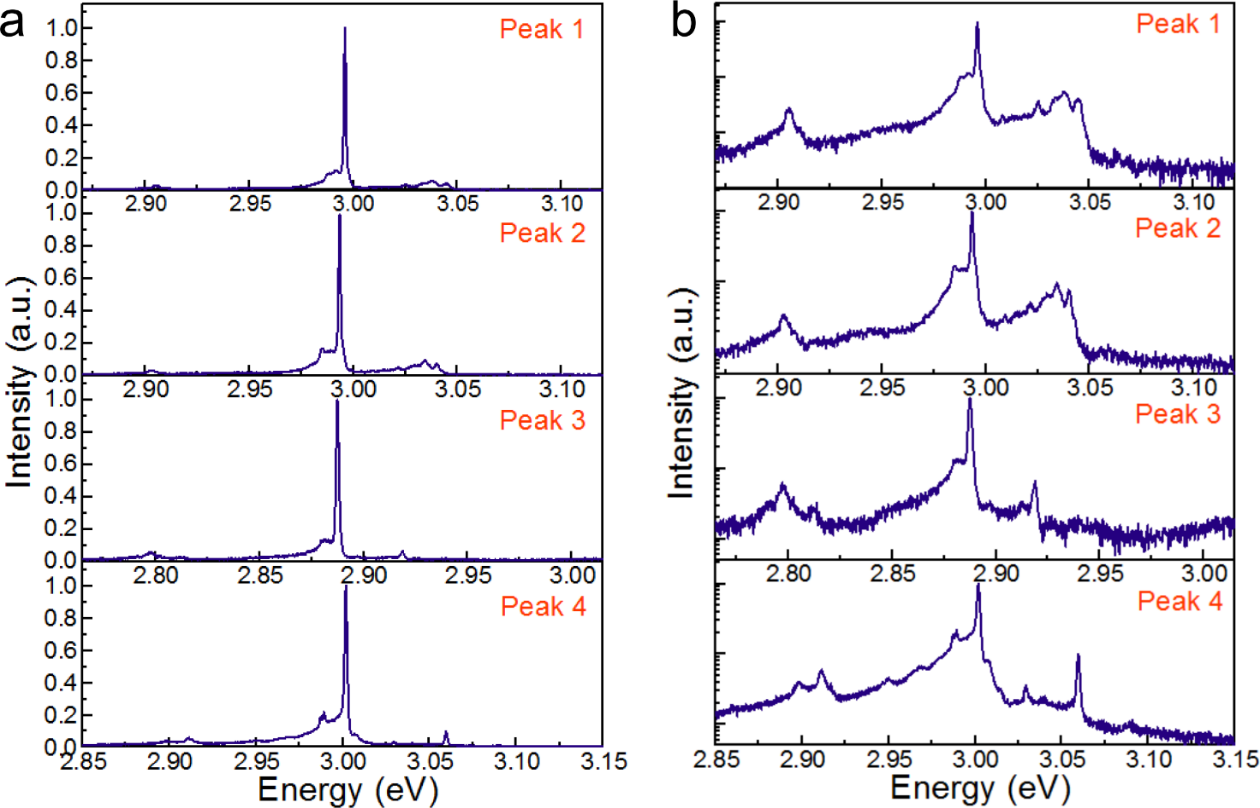


**Figure S2.** Typical PL spectra in linear scale a) and in log scale b) measured from emitters in our sample. Four examples of emitters with zero phonon lines at 2.99 eV, 2.99 eV, 2.89 eV and 3.00 eV.

1. **Power dependence of a typical emitter**

The intensities of all peaks from a typical emitter below saturation grow linearly with excitation power, indicating their excitonic, rather than biexcitonic nature.


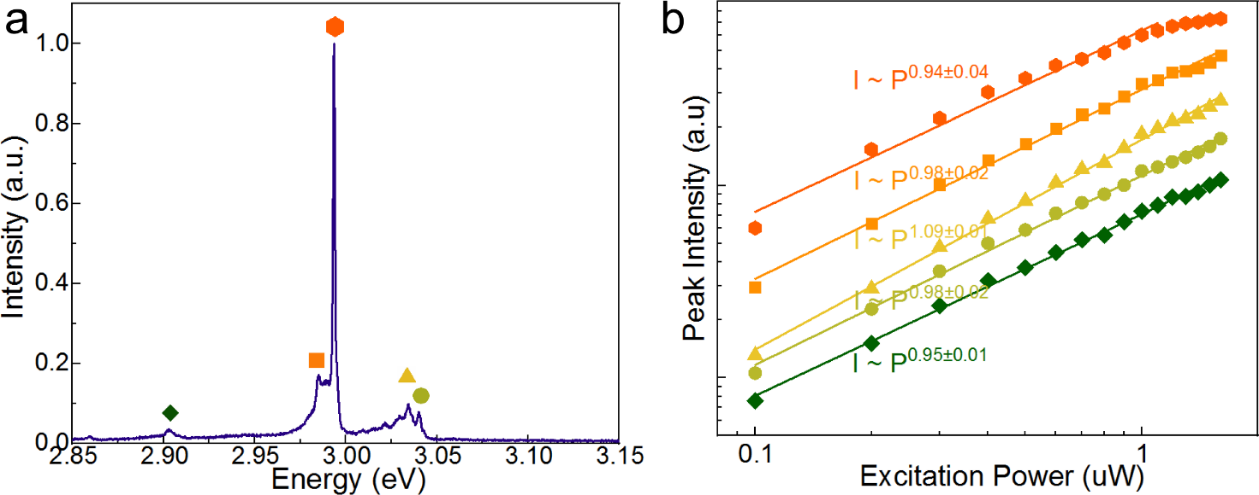


**Figure S3.** a) A typical emission spectrum measured at 8 K. b) The power dependences of the integrated intensity of the peaks labelled in a).

1. **Exciton lifetimes as a function of temperature**

The emission lifetime decreases monotonically with increasing temperature, as emission intensity decreases. This decrease in emission time, along with the rapid quenching of the emission intensity is evidence of increased non-radiative recombination. The fact that the lifetime does not exhibit any clear saturation at low temperature reveals that non-radiative processes are likely occurring even at temperatures below 10K.


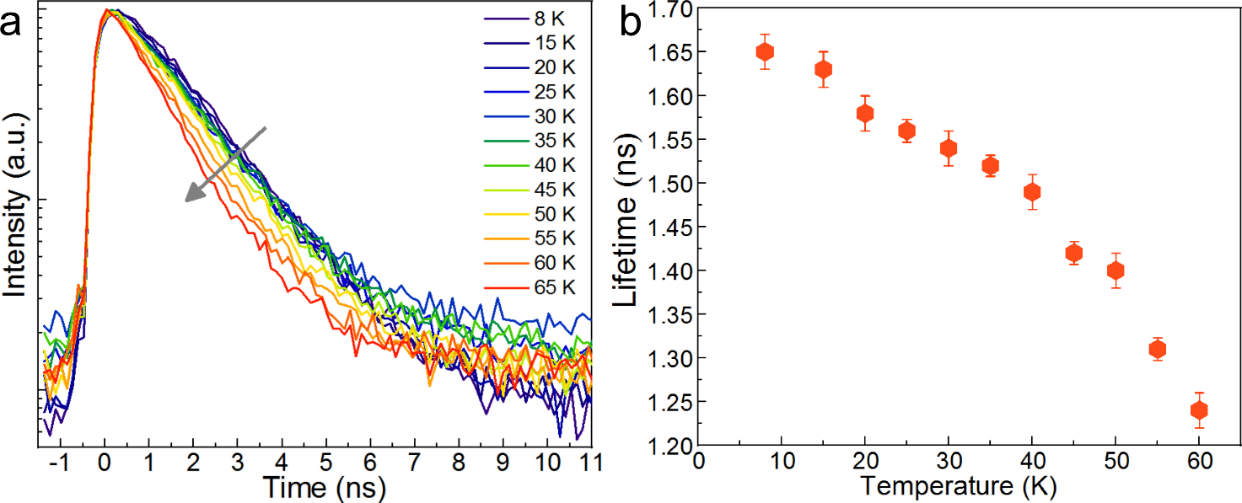


**Figure S4.** a) Temperature-dependent PL decay excited by a 355 nm laser. b) The extracted effective PL lifetime.

1. **Polarization behavior of the emitter**

A half wave plate and a polarizer were used to rotate the polarization and filter the single photon emission. As a result, the polarization profile for this representative emitter is shown in Figure S5. The emission polarization visibility is calculated using the intensity contrast equation:

$I= \frac{I_{max}-I_{min}}{I_{max}+I_{min}}$ (1)

where $I_{max}$ and $I_{min}$ are the maximum and minimum intensities at different angular directions of the polarizer, yields the value for emission polarization visibility of ~30%. Notably this emission is not fully polarized, being different from most other reported polarization behavior of SPEs. We suggest this phenomenon is due to the fact that our emitter being isoelectronic center and that would most likely be deep level defect in GaN matrix. By definition, a deep center implies a localized potential to bound exciton, while their wavefunction is spread in the momentum space. The property of this kind defect is thus not the same as that of shallow defect, having complex wave function and probably won’t follow the behavior of the band edge.

**
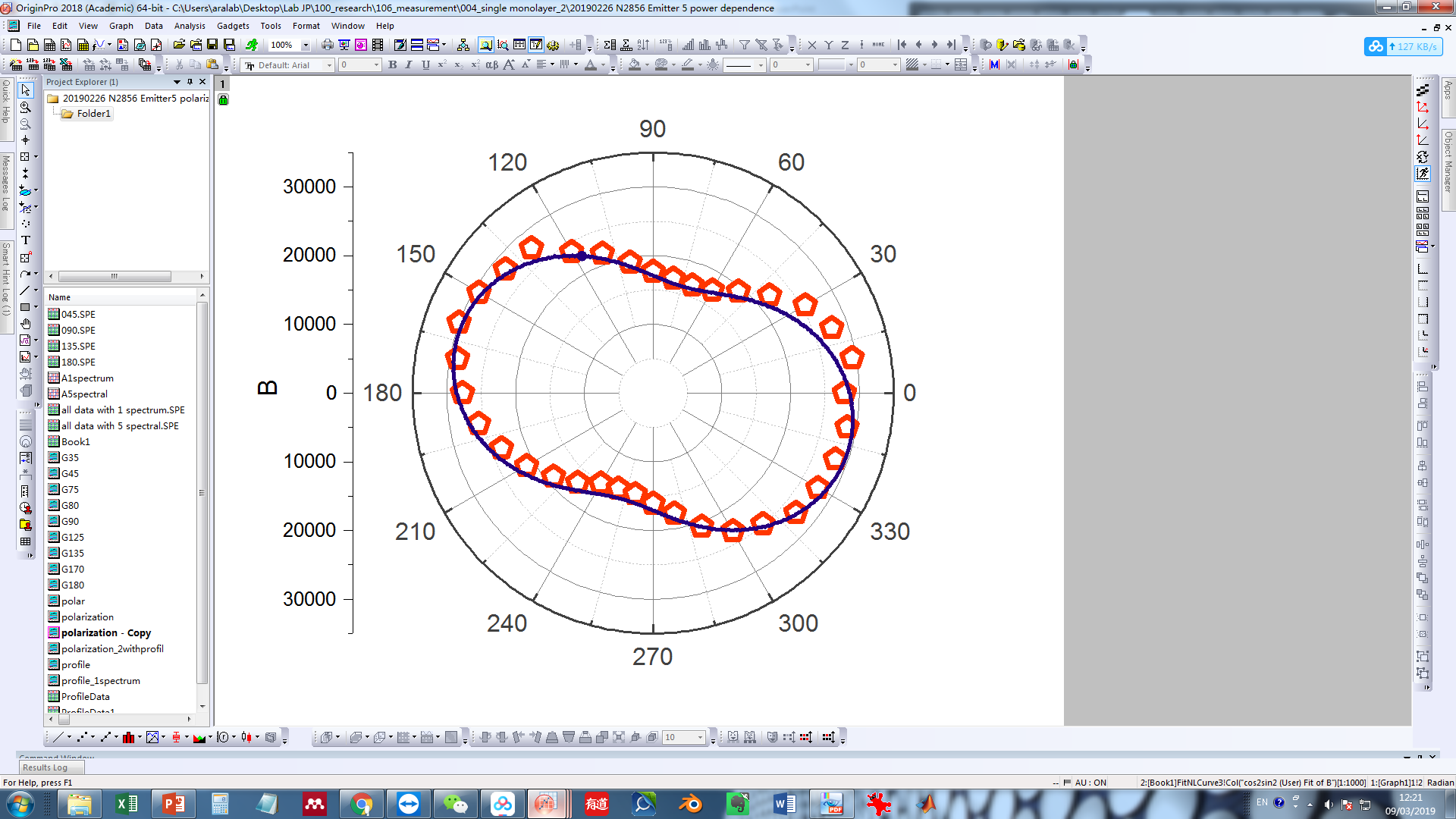
**

**Figure S5.** Polarization measurement of the emission (red open pentagon).

1. **Photon extraction efficiency**

FDTD simulations of the emission profile of an emitter in an etched nanowire. 8% of the light is collected into a numerical aperture of 0.4.


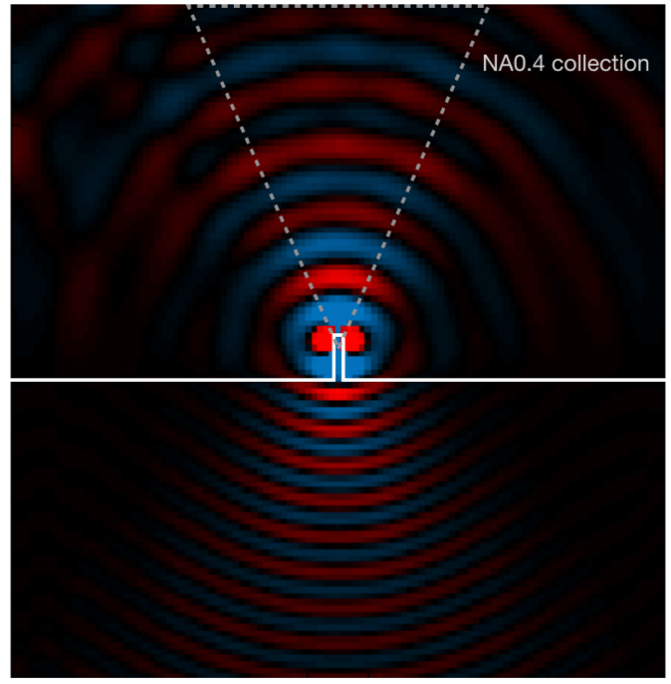


**Figure S6.** FDTD simulations of the emission profile of an emitter in an etched nanowire.
